# *In Vivo* Models and *In Vitro* Assays for the Assessment of Pertussis Toxin Activity

**DOI:** 10.3390/toxins13080565

**Published:** 2021-08-12

**Authors:** Marieke Esther Hoonakker

**Affiliations:** Institute for Translational Vaccinology (Intravacc), P.O. Box 450, 3720 AL Bilthoven, The Netherlands; Marieke.Hoonakker@intravacc.nl

**Keywords:** pertussis toxin, acellular pertussis vaccines, *in vitro* assays, *in vivo* models

## Abstract

One of the main virulence factors produced by *Bordetella pertussis* is pertussis toxin (PTx) which, in its inactivated form, is the major component of all marketed acellular pertussis vaccines. PTx ADP ribosylates Gα_i_ proteins, thereby affecting the inhibition of adenylate cyclases and resulting in the accumulation of cAMP. Apart from this classical model, PTx also activates some receptors and can affect various ADP ribosylation- and adenylate cyclase-independent signalling pathways. Due to its potent ADP-ribosylation properties, PTx has been used in many research areas. Initially the research primarily focussed on the *in vivo* effects of the toxin, including histamine sensitization, insulin secretion and leukocytosis. Nowadays, PTx is also used in toxicology research, cell signalling, research involving the blood–brain barrier, and testing of neutralizing antibodies. However, the most important area of use is testing of acellular pertussis vaccines for the presence of residual PTx. *In vivo* models and *in vitro* assays for PTx often reflect one of the toxin’s properties or details of its mechanism. Here, the established and novel *in vivo* and *in vitro* methods used to evaluate PTx are reviewed, their mechanisms, characteristics and limitations are described, and their application for regulatory and research purposes are considered.

## 1. Introduction

Whooping cough, or pertussis, is caused by infection with the Gram-negative bacterium *Bordetella pertussis.* Although people of all ages are susceptible to pertussis infection and can transmit the disease, its impact on health is most severe in young children and infants. Despite intensive vaccination programs, there remains an estimated 24 million cases and 160,700 deaths from pertussis each year [1]. The first generation of pertussis vaccines were developed in the 1920s and were made of bacteria which were killed by exposure to inactivating chemicals and heat. Although these whole-cell pertussis (wP) vaccines have proven to be highly effective, and are still used in many countries, they can cause fever, malaise, and pain at the injection site. Due to these side effects from the wP vaccines, parents are frequently reluctant to have their infants and children receive the required booster doses, thereby impacting vaccination coverage. To alleviate some of these problems, a second generation of pertussis vaccines was developed in the 1970s which contained a minimum of purified bacterial components, considered important for inducing protective immunity. The acellular pertussis (aP) vaccines all include inactivated pertussis toxin (referred to as pertussis toxoid (PTd) when inactive), and a combination of other pertussis virulence factors such as filamentous hemagglutinin (FHA), pertactin, and/or fimbria types 2 and 3. aP vaccines are the vaccine of choice in most high income countries and are considered less reactogenic than wP vaccines, which are still widely used in other parts of the world, including Latin America, Africa, and parts of Asia [2].

The inclusion of PTd is considered essential for aP vaccine-induced protective immunity [3,4], but its manufacture must be carefully controlled in order to ensure the sufficient chemical inactivation of pertussis toxin (PTx) without compromising the quality of its antigenic epitopes. With insufficient chemical inactivation, there is a risk of residual activity, but it would conserve those epitopes that confer protective immunity; whereas, excessive inactivation eliminates the risk of residual PTx activity but yields antigens that may not induce protective immunity. The balance between these two attributes (residual activity and protective antigens) is critical in the manufacture of safe and effective vaccines and is, therefore, an important concern in regulation and batch release testing. In vitro and *in vivo* methods developed for the assessment of PTx activity in aP vaccines have recently been reviewed by others [5]. In addition to safety testing, PTx is also used in toxicology research, cell signalling, research involving the blood–brain barrier, and testing of neutralizing antibodies. Therefore, this review focusses on assays used for the determination of residual PTx activity in the context of aP vaccines and purified PTx, with an emphasis on assay mechanisms and characteristics, their limitations, and the regulatory considerations for adapting the methods for regulatory and non-regulatory purposes.

### 1.1. PTx Structure, Function and Biology

**Figure 1:**
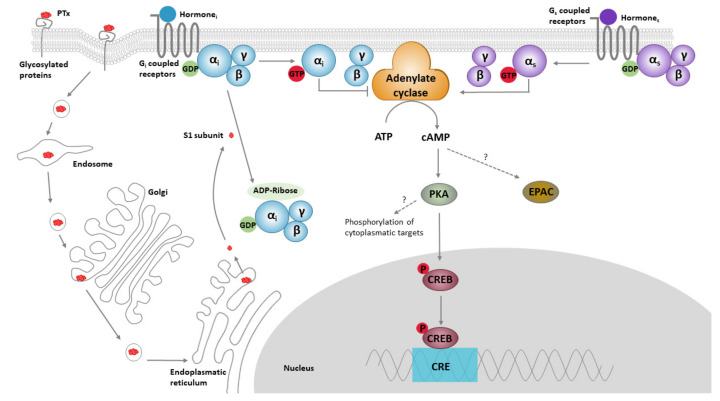
The classical models for PTx binding, internalisation, ADP ribosylation, and its effect on cell signalling. The B oligomer of PTx binds to glycoconjugate proteins on the cell surface, upon which the holotoxin enters the cell by endocytosis, followed by retrograde transport through the endosome, Golgi and the endoplasmatic reticulum. Subsequently, the S1 subunit is released into the cytosol. Within the cytosol, the S1 subunit catalyses the transfer of ADP-ribose from NAD^+^ to the α-subunit of Gα_i/o/t_ proteins, thereby preventing interaction of these proteins with their cognate receptors. ADP ribosylation fixes the α-subunit of the G-proteins in their inactive (GDP-bound) form, thereby rendering it unable to inhibit its target enzymes; ACs. ACs catalyse the conversion of ATP into cAMP. This second messenger binds to and activates protein kinase A (PKA), which is involved in a range of pathways, one of which is the phosphorylation of the cAMP response element-binding protein (CREB). CREB binds to the DNA sequence cAMP response elements (CRE) and thereby increases the transcription of CRE responsive genes. PTx-induced cAMP might also directly bind to “exchange protein directly activated by cAMP” (EPAC), which are guanine nucleotide exchange factors for Rap molecules.

PTx is an AB-type bacterial toxin composed of an A-protomer made of the S1 subunit, and a B-oligomer comprised of subunit proteins S2 to S5. The B-oligomer complex is responsible for binding of the toxin to glycosylated proteins on cell membranes. Upon binding, the toxin is internalized and transported in a retrograde manner through the Golgi complex and endoplasmatic reticulum. Once in the endoplasmic reticulum, the S1 subunit dissociates from the B-oligomer and is released into the cytosol [6], where it hydrolyses NAD and transfers the released ADP-ribose to the α subunit of inhibitory/other/transducin G (Gα_i/o/t_) protein-coupled receptors [7,8]. This prevents GDP to GTP exchange and fixes the Gα_i/o/t_ subunits in a dissociated and inactive GDP-loaded state. Consequently, the inactive Gα_i/o/t_ subunits cannot inhibit their target enzymesadenylate cyclases (AC), or affect other target proteins. This potentially causes an increase in the second messenger cAMP, and subsequent downstream effects (Figure 1). This mechanism of PTx action is considered to be the cause of the majority of its biological effects, including exocrine secretion, lipolysis, stimulation of pancreatic islet cells, histamine sensitization, leukocytosis, and inhibition of lymphocyte and neutrophil migration [9,10]. However, PTx-dependent ADP ribosylation might also affect various other cellular mechanisms such as the expression of α_i_ and α_o_ subunits [11,12,13], regulation of PI3K/Akt pathway [11], tuberin phosphorylation, and RSG expression [12], as well as ERK/MAP kinase pathways [13,14]. In addition, various ADP ribosylation independent effects of PTx on cell signalling have been described and include the ERK/MAP kinase pathway [15,16,17], and pathways involving tyrosine kinase [18], NF-κB [19] and Rac [20]. Other non-cAMP mechanisms related to the binding of PTx to receptors include the proliferation of T cells [15,16], glucose oxidation in adipocytes [16], and activation of TLR4 [19]. 

### 1.2. Role of PTx in Pathology and Toxicity

There is some debate regarding the role of PTx in the pathology of pertussis disease. Several lines of evidence underline the importance of PTx for some aspects of the disease such as leukocytosis and the paroxysmal cough [9]. However, despite its name as a toxin—and unlike many other bacterial toxins—PTx is not considered to be particularly noxious or harmful to humans and animals (Table 1). Indeed, considering its high potency to ADP-ribosylate Gα_i_ proteins, the *in vivo* response to PTx is rarely one of distress, morbidity or mortality. For example, in the mouse histamine sensitization test described below, the apparent health and behaviour of the animals is normal for several days after PTx administration. In that test, it is not the PTx to which the animals succumb, but rather the histamine administration at a dose which would otherwise be safe in mice not exposed to PTx. In humans, the acute LD_50_ of PTx has never been estimated and it is impossible to extrapolate an unsafe amount of PTx based on animal data due to the inter-species differences. Nevertheless, a large dose of 0.5 or 1.0 μg/kg of purified PTx has been administered intravenously to adult volunteers in order to study its effect on insulin and blood glucose levels and these volunteers did not experience any reported adverse outcomes [21]. Although the prior immune status or history of vaccination to pertussis was not provided, this study suggests PTx doses in the ranges administered in the study are not toxic for adults. However, it should be kept in mind that the disease caused by *B. pertussis* can be extremely different in infants under three months of age and is associated with complications such as pulmonary dysfunction and hypertension, leukocytosis, and encephalopathy [22]. As the immune status and the function of the immune system of these extremely young individuals differ from adults [23], the interpretation and extrapolation of the data from studies with adult subjects should be done with care.

### 1.3. PTx Reference Preparations

The diverse properties of PTx require different approaches and methods, as is reflected in the particularly diverse *in vivo* models and *in vitro* assays that are addressed in this review. All of the methods have their own characteristics and limitations, and the methods cannot easily be compared due to their diverse nature, and as a result of the different PTx preparations used across laboratories. A comparison is further compounded by the specific activity of each PTx preparation which, like any purified enzyme, is not directly related to their stated protein content (see Table 2). While early studies utilized PTx purified in house, subsequent studies often included an international reference preparation. The JNIH-5 international reference standard was prepared in 1984 and assigned an activity of 10,000 IU (International Units) per vial for the *in vivo* histamine sensitization test and the *in vitro* CHO (Chinese hamster ovary) cell clustering assay (both discussed in more detail below) [21]. However, at the time of calibration, the CHO cell clustering assay was not standardized between laboratories, thereby resulting in a high variability of reported PTx activities. Subsequent calibration studies using standardized protocols caused a diversification between the CHO and HIST unitage. Due to shortage on both the JNIH-5 and the Biological Reference Preparation 1 (BRP1), international collaborative studies were performed to determine the suitability of the 2nd WHO International Standard (IS) for Pertussis Toxin (coded as 15/126) and the replacement preparation BRP2. The 2nd IS reference preparation was assigned 1881 IU/ampoule in the HIST and 680 IU/ampoule in the CHO cell clustering assay [29]. The BRP2 was only calibrated in the CHO cell clustering assay and assigned 130 IU/vial [30]. This highlights the importance of calibrating PTx preparations, and also shows that the units of activity might differ between methods and can thereby influence the interpretation of results.

## 2. *In Vivo* Models

### 2.1. Histamine Sensitization Test (HIST)

**Figure 2:**
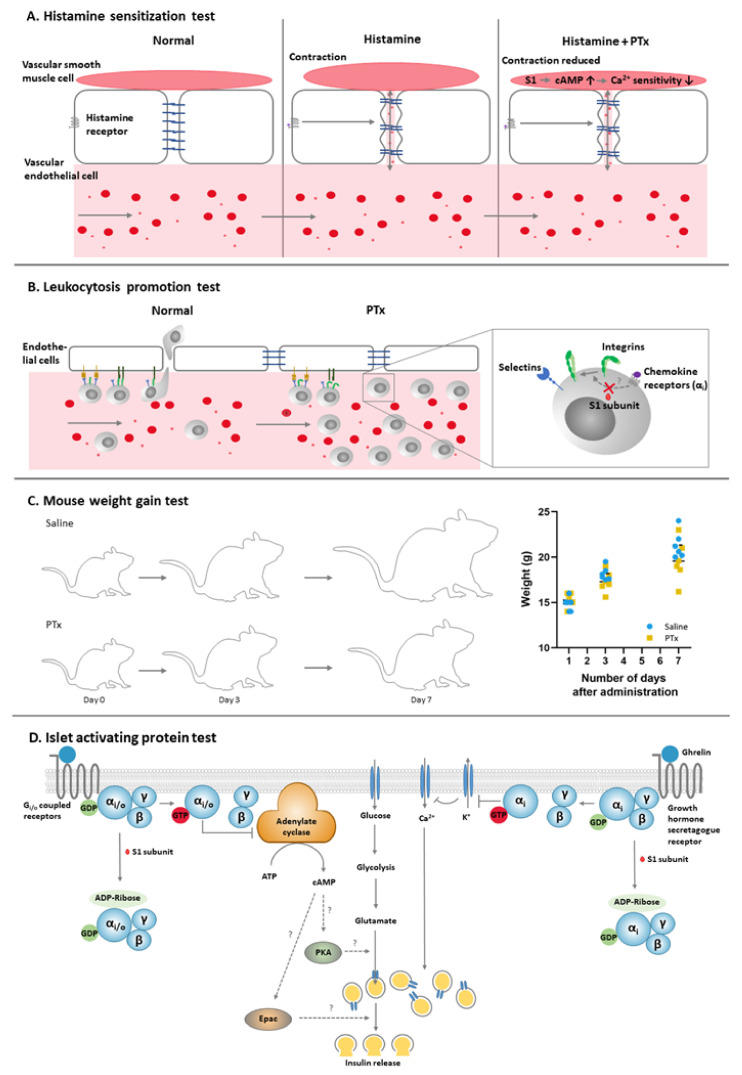
The classical *in vivo* methods for PTx and models for the mechanisms. (**A**). The histamine sensitization test is based on the sensitizing effects of PTx in mice to histamine doses which would normally cause no effect. Under “normal” conditions, vascular permeability and blood pressure is maintained by the vascular endothelial cells and vascular smooth muscle cells. Administration of histamine results in the reorganization of actin filament structure and adherence junctions, increased endothelial permeability and vascular leakiness. Contraction of vascular smooth muscle cells can (partly) compensate for the blood volume loss, but this compensatory mechanism is inhibited by PTx, causing a significantly reduction in blood pressure. (**B**). In the leukocytosis promotion test, the effect of PTx on leukocyte numbers is measured. PTx inhibits lymphocyte extravasation and restores lymphocyte egress from lymph nodes to the lymph, resulting in a rise in circulating leukocyte number. Migration involves rolling mediated by attachment to selectins and arrest mediated by integrins. The binding of integrins and endothelial adhesion molecules triggers the opening of endothelial junctions, allowing leukocytes to transmigrate. PTx does not affect selectins and rolling, but does inhibit integrin mediated arrest. Integrins can only bind to adhesion molecules on the high endothelial venule cells upon activation by Gα_i_-coupled chemokine receptors. These Gα_i_-coupled receptors might be the target of PTx, although additional research will be necessary to confirm whether these receptors are responsible for the leukocytosis. (**C**). The mouse weight gain test is based upon the negative effect of PTx on the weight gain of mice throughout a period of seven days. Although considered a general toxicity test, its mechanism is unknown. (**D**). In the islet activation protein test, PTx enhances the glucose-induced release of insulin. In the test, PTx is responsible for ADP ribosylation of Gα_i/o_ proteins in β-cell, resulting in accumulation of cAMP. Although not studied directly in relation to PTx, enhanced cAMP levels can activate PKA and EPAC and stimulate the glucose pathway that induces the release of insulin. The other pathway shown to be involved in the IAP test is mediated by ghrelin. Ghrelin is an endogenous ligand for the Gα_i_-coupled growth hormone secretagogue receptor, which can suppress K^+^ exflux. As a result of the ADP ribosylation of this Gα_i_-coupled receptor, K^+^ exflux is inhibited. The resulting depolarization causes Ca^2+^ influx and enhanced levels of Ca^2+^ are essential for the release of insulin.

The murine histamine sensitization test (HIST) was one of the first *in vivo* methods used to study the biological effects of PTx. It is based on the discovery in 1948 by Parfentjev and Goodline that PTx sensitizes mice to histamine. Whereas mice are normally tolerant to the effects of histamine, their pre-treatment with PTx reduces the histamine lethal dose 30–300 times [31,32]. Although the biochemical mechanism by which PTx induces histamine sensitization has not been fully elucidated, much of it is well understood (Figure 2). The process is likely initiated by the ADP ribosylation of Gα_i/o_, as an active S1 subunit of PTx is known to be required for its HIST activity [33]. Increased permeability of blood vessels occurs as a results of PTx treatment and has been suggested to underly histamine sensitization [22]. Adrenalectomy has a similar effect, resulting in vascular fluid loss and sensitization to histamine, an effect that can be counteracted by injection of compensatory volumes of saline [32]. Additional evidence for the central role of the vascular system in histamine sensitivity comes from studies that demonstrate that PTx affects the contractile properties of arteries and results in hypersensitivity to histamine-induced vasodilation [23,32]. A similar effect is also observed after removal of adrenal glands and after blocking of β-adrenergic receptors of mice, suggesting a common catecholamine-dependent mechanism [34]. The effect of PTx on the vascular system is further supported by its capacity to reduce the blood pressure of spontaneous hypertensive rats [35,36,37,38]. These lines of evidence strongly point toward the central role of dysregulated arteries in the PTx-induced sensitization of mice to histamine.

Due to the high potency of PTx in the HIST, this model can be used to detect low levels of active PTx, such as residual levels which may be present in aP vaccines. In this capacity, the HIST has been adopted by manufacturers for stability monitoring, process optimization, routine batch release testing of aP vaccines to confirm the absence of residual PTx activity [39], and to ensure consistent PTx activity in wP vaccines [31]. The test has also been applied for other PTx-related research purposes, such as the evaluation of neutralizing antibodies [40,41,42].

The basic protocol for conducting the HIST as a batch release test for aP vaccines involves the administration to mice of either the vaccine under study, or a positive (purified PTx) or negative (saline) control and followed 4–5 days later by an injection with histamine. Death of the mice within 24–48 h, or a reduction in body temperature following the histamine injection indicates the presence of active PTx in the vaccine. The requirements and interpretation of the HIST as a vaccine batch release test vary greatly between regulatory regions. Differences in the protocols include the mouse requirements (sex, age, strain, etc.), inclusion of positive and/or negative controls, number of animals per test group, dose of histamine and type of histamine salt, the interval between vaccination and challenge, and the interval between challenge and test outcome [5,39,43]. As a result of these differences, the comparison of the data between laboratories is often difficult, if not impossible. This problem is further compounded by the inherent variability of the method, even within an established laboratory, as well as the use of different PTx preparations. However, the inclusion of PTx reference preparations calibrated against an international standard does amend some of these issues. 

Apart from these potential protocol differences there are three distinct versions of the HIST. The first version determines if the PTx content is at or below an acceptable threshold based on the number of mice succumbing as a result of the histamine challenge [39,43]. The other two versions are very similar and assessment of the PTx content is based on body temperature change measured shortly after the histamine administration [44], either as a pass/fail test or as a quantitative test which measures the amount of PTx by comparison to a reference preparation [45]. Assessment of the body temperature can be done using a rectal probe or by measuring the dermal temperature with an infrared camera [46]. No clear correlation has been observed between the temperature method and the lethal method endpoints [46], suggesting potential differences in their downstream mechanisms of action. The reported sensitivities of the methods for various PTx preparations are listed in Table 3.

The HIST remains the most widely recognized method for the quality control and regulation of aP vaccines and other PTx related research purposes. However, over the past two decades the HIST has faced increasing criticism. The test is associated with serious animal welfare concerns due to the anaphylactic shock, drop in body temperature, pain, distress, and death it causes to the mice. The high variability of the method also poses an unnecessary risk of delays to product release due to re-testing requirements or possible loss of a vaccine batch that would otherwise be considered safe. The lack of correlation between the increased sensitivity to histamine in mice and the potential adverse effects in humans, also brings into question the utility of the HIST method. Some, if not most, regulatory authorities interpret the HIST as an indicator of vaccine safety, whereas others view it as a means to monitor the consistency in the PTx inactivation process. This difference in regulatory interpretation can impact the ease by which alternative methods to the HIST may be adopted for use in the quality control of aP vaccines. In regions where an animal test for safety is required, the regulatory adoption of an alternative method to the HIST could be lengthy and difficult; however, national regulations encouraging 3Rs principles would facilitate this process. Where an animal safety test is not required for batch release purposes and the HIST is used as an indicator of product consistency, there is growing pressure to discontinue the HIST and implement suitable *in vitro* methods, such as those described in the following sections. As such, the European Pharmacopeia (Ph. Eur.) removed the requirement to test the PTx content in the formulated end product and the test for reversion of PTd, provided that the PTx content of the bulk product is tested *in vitro* and a history of safe use and PTd stability can be demonstrated.

### 2.2. Leukocytosis Promotion (LP) Test

The leukocytosis promotion (LP) test is based on the original observation from 1900 that pertussis infection is associated with an increase in leukocyte numbers, although this increase is more pronounced in children and occurs early during the infection period [53]. More recent studies confirm that hyperleukocytosis frequently occurs in infants that suffer from pertussis and is associated with a poor prognosis; however, it is unclear whether leukocytosis contributes to the disease symptoms or is just an indicator of poor outcome [54]. In infants, leukocytosis does not occur upon infection with *B. parapertussis,* which lacks PTx [55], or upon infection with a PTx-deficient *B. pertussis* strain [56], thereby suggesting that PTx is the agent responsible for this biological effect. In addition, PTx-neutralizing antibodies significantly reduce leukocytosis upon *B. pertussis* infection in baboons and mice [57]. Leukocytosis following the administration of PTx has also been observed in rats [58], pigs [59], and macaques [60,61].

When used as a vaccine batch release test, leukocyte counts are determined in mice three to six days post injection with PTx or the vaccine under study. This *in vivo* model can detect a range of 20–4000 ng of PTx [48], although its sensitivity depends on the route of injection [24]. Several studies demonstrate that PTx inhibits lymphocyte extravasation [62] and restores lymphocyte egress from lymph nodes to the lymph [63], the first process depending on Gα_i_ protein-linked signalling [62,64]. Although this partly reveals the mechanism of the LP response (Figure 2), much of the details remain unclear.

As with the HIST, there are several protocols for the LP test which vary with regard to the route of administration, the interval between administration and blood sampling, the mice strain used, and the number of mice per group [24]. Although these differences add to the variability associated with the LP test results, the method is considered to be less variable than that observed for the HIST and has less impact on animal welfare [24]. The LP test is included in the WHO Manual for Quality Control of Diptheria, Tetanus, and Pertussis Vaccines as an acceptable test for the control of wP vaccines [65], but not for aP vaccines [66], and is also described as a release method for aP vaccines in the Japanese pharmacopeia.

### 2.3. Mouse Weight Gain (MWG) Test 

The mouse weight gain (MWG) test was developed in 1965 by Pittman and Cox [67], and is based on the observation that the administration of wP vaccines reduced the weight gain of mice. The outcome of the MWG test has been associated with the occasional reactogenicity of wP vaccines in children [68,69,70,71,72,73,74,75], and therefore the MWG test is recommended in the routine lot release testing of wP vaccines by WHO and the Ph. Eur. [76]. In contrast to the LP test, the MWG test can be considered as a general toxicity test as the reduced weight gain can also occur as a result of heat-labile (dermonecrotic) toxin, lipopolysaccharide (LPS), adenylate-cyclase toxin, and tracheal cytotoxin [24] which may be present in wP vaccines. In the test, the vaccine or agent under study is administered to mice and the weight gain of these mice is compared to the weight gain of control animals three- and seven-days post-administration (see Figure 2). As a similar route of injection and volume are used in the MWG test and the LP test, these *in vivo* models are often combined, thereby reducing the required number of animals.

Although the MWG test was originally developed for wP vaccines, it has been used in the analysis of individual virulence factors and for the testing of aP vaccines. However, the injection of purified PTx in mice has had contradictory impacts on the weight gain of mice. Where two studies reported 0.45 µg and 4 µg PTx to enhance the weight gain [40,41], another reported reduced weight gain following the administration of 375–1500 ng PTx [50]. Another study demonstrated that the administration of a safe aP vaccine did not significantly affect the weight gain of mice, whereas an aP vaccine estimated to contain 2–6 HIST units (Japanese reference preparation), did increase the weight gain [41]. The variability in these results might be a reflection of the dose or specific activities of the various sources of PTx used in these studies.

As with the other animal models, various aspects of the MWG test differ between laboratories, making a comparison of the results difficult. The differences include the mouse strain, sex, number of animals per group, and the dose ranges tested [24]. The application of the MWG test for both research and regulatory purposes might be difficult as the direction of the weight gain change may depend on the protocol used and the activity of the used PTx preparation. Application is also compounded by the incomplete understanding of the model’s mechanism of action and by the intra- and interlaboratory variability in results. However, careful inhouse validation and justification of the method may allow for its utility for quality control and research related purposes. 

### 2.4. Islet-Activating Protein (IAP) Test

The islet-activating protein (IAP) test is based on an observation by Regan and Tolstoouhov that children suffering from a *B. pertussis* infection tend to have slightly reduced glucose levels, despite eating a normal diet. Rabbits and mice infected with *B. pertussis* were similarly reported to develop hypoglycaemia [77,78] and mice and rats vaccinated with wP vaccines also experienced hypoglycaemia and hyperinsulinemia [79,80]. An early study by Toyota et al. showed that PTx normalized the response to glucose in spontaneous diabetic rats for a month [17], and PTx was therefore evaluated as a treatment for diabetes in humans [17].

Islets of Langerhans normally release insulin at a slow rate, a process that is catalyzed by glucose exposure. Epinephrine induces a slight decrease in insulin levels, but causes a steep increase in insulin levels in rats pre-treated with PTx [79]. Based on these observations, rats were treated with purified PTx followed by the administration of glucose prior to the collection of blood samples [42]. This resulted in hyperinsulinemia for several weeks post PTx administration. The procedure formed the basis for the IAP test where rats receive an intravenous injection with a range of PTx concentrations or the preparation under study and are then challenged with glucose three days later. The level of glucose and insulin are assessed in blood samples taken immediately before the challenge and 15 min afterwards. Using this procedure, increased insulin secretion can be detected three to ten days post administration of PTx in a dose-dependent manner with a range between 8 ng and 2 µg/dose [42]. 

Experiments using islets isolated from rats [81] and mice [82] were performed to investigate the mechanisms underlying the effect of PTx on insulin secretion. Islets isolated from PTx treated mice were associated with lower blood glucose levels and enhanced insulin levels [82]. Similarly, islets from PTx-treated rats released higher levels of insulin than control islets and negated the inhibitory effect of epinephrine [72]. PTx has a similar effect on islets from untreated rats [73] and mice [82], that upon exposure to PTx release insulin in a PTx concentration-dependent manner (0.001–1000 ng/mL)[73]. PTx was not found to affect any properties of ACs including their affinity for substrates, sensitivity to guanine nucleotide or fluoride activation, thereby suggesting that neither ACs nor the Gα_s_ is the target site of PTx in islets [74]. This was confirmed by a study showing that PTx specifically ADP-ribosylated a protein of 41 kDa in the islets of membrane of PTx treated rats [75], which corresponds to α_i/o_ subunits [83]. Although not directly studied in relation to PTx, increased cAMP levels and stimulation with glucose have been shown to be essential for insulin release [84]. The rates of ADP ribosylation by PTx differed among the different G-proteins, Gα_o_ being affected first, followed by Gα_i2_ and Gα_i3_ [72]. These observations strongly suggest that the effect of PTx on islet function depends on the ADP-ribosylation of α-subunits. Therefore, although the IAP test has not been part of official regulatory requirements for the detection of PTx, it is a well-studied and substantiated method and could be a valuable method in the further studies on PTx activity.

### 2.5. Other In Vivo Models

As PTx has been used in a large range of research areas, many *in vivo* models have been developed to further evaluate its effects and mechanisms of action. Although these *in vivo* models are only suitable for specific purposes, they provide important insight into certain properties of the toxin. As discussed above, PTx affects *in vivo* vascular permeability, a feature which has been visualized by a model developed by Sukuma et al. [51]. In this model, extravasation is monitored by intracutaneous injection of PTx combined with an intravenous administration of a blue dye in either rabbits or guinea pigs. As a result of the extravasation, the dye spreads subcutaneously and the area of the staining is correlated with the concentration of PTx. This model has been demonstrated to have a sensitivity to PTx between 1 ng and 100 µg per dose [51]. However, the extravasation of the dye was observed in the animals following injection with native PTx as well as with PTx heated to 56 °C, thereby suggesting the model might not allow for discrimination between PTx and a thermal-denatured PTd. Comparison of PTx and the non-catalytic mutant PTx revealed that both increase vascular permeability [52], suggesting an ADP-ribosylation independent mechanism. This model has been applied for research purposes, e.g., to evaluate the PTx neutralizing mechanism of action of a set of monoclonal antibodies [85] but has not been used for quality control or routine vaccine batch release purposes.

The role of PTx in the modulation of the innate immune response has been investigated extensively *in vivo*, primarily using mice and baboon models. These studies indicates that PTx binds to TLR4, upregulates P-selectin [86], and affects the function of resident alveolar macrophages [87], dendritic cells [88], migration of macrophages into the peritoneal cavity [89], and recruitment of neutrophils [90,91,92]. PTx can also affect adaptive immune responses and contribute to Th1 and Th2 CD4^+^ T cells responses [93], aid Th17 differentiation [94,95], trigger the activation of CD8^+^ T cells [96], and support B cell migration from the bone marrow [97]. PTx has also been widely used as a means to facilitate the onset of experimental autoimmune encephalomyelitis (EAE) in animal models of human multiple sclerosis (MS). EAE is commonly achieved by injecting myelin protein(s) or peptide(s) in combination with (incomplete) Freund’s adjuvant, generally followed by injection(s) with PTx [98,99,100]. This overcomes the tolerance against the autoantigens and promotes migration of immune cell into the central nervous system, resulting in MS-like symptoms such as paralysis of specific parts of the body. Interestingly, repetitive exposure to low doses of PTx prior to immunization with myelin peptide protects mice from developing EAE and enhances the number of regulatory T cells [101]. Similarly, a large dose of PTx in an EAE model substantially attenuates the demyelination and severity of symptoms [102]. PTx has also been studied for its effects on tumor cells and the related immune responses. Rat models showed that the administration of PTx substantially reduced tumor growth and the number of regulatory T cells [103] and improved the survival rates [104]. Studies in rats and mice [90,105] and in humans have suggested PTx may be a potential therapeutic agent for the treatment of bladder cancer. As cell migration and invasiveness are strongly correlated with the malignant properties of this tumor type, *in vitro* studies were performed and showed that PTx (0.01 ng/mL) ADP-ribosylated Gα_i_-coupled receptors in the bladder cancer cell line J82, and inhibited the migration of the cells toward lysophosphatidic acid [91]. In addition, a rat and mouse model based on chemically induced bladder carcinoma demonstrated that intravesical administration of PTx reduces tumor development [90,105] A phase I study showed that application of PTx to patients suffering from bladder cancer tolerated the infusion of up to 72 µg PTx into the bladder [105]. Despite these positive results, additional research will be necessary to confirm the possible therapeutic application of PTx.

### 2.6. Ex Vivo Methods

PTx also affects ex vivo cultured erythrocytes, platelets and adipocytes. Similar to FHA of *B. pertussis*, PTx triggers the agglutination (clumping) of erythrocytes [92]. The capacity of FHA and PTx to induce agglutination have been studied in various species, revealing that FHA is more potent than PTx [92]. Nevertheless, PTx induces agglutination of many species, but most potently affects the erythrocytes of geese, horses, and rabbits. The agglutination of erythrocytes was found to be an all-or-nothing event and occurred between 8 and 1000 ng/mL of PTx [106,107]. Two studies suggest that the agglutination is a PTx B oligomer-dependent phenomenon, as it is induced by both the enzymatic-inactive PTx mutant [108] and the B oligomer itself [109]. Assays of agglutination have been used to study the neutralizing properties of monoclonal antibodies and antisera using geese [106,107,110] and chicken [3] erythrocytes, but might also be considered for other PTx related purposes.

Another attribute of PTx is its capacity to induce aggregation of platelets, which is considered a B oligomer-dependent characteristic. The B oligomer has been reported to activate human platelets in a concentration-dependent manner (1–10 µg/mL) [98] and thereby suggests that is might be a valuable method to study the B oligomer specifically. Its effect on platelets has been determined by intracellular calcium concentration, granule secretion, and aggregation [98,99].

Early studies by Endoh et al. [100] and Garcia-Sainz et al. [111] showed that PTx causes lipolysis in cultured adipocytes of rats and hamsters even at PTx levels as low as 1–100 ng. Exposure of adipocytes to PTx resulted in the generation of cAMP and reduces their sensitivity to adenosine inhibition [112,113]. Although these studies suggest an AC-dependent mechanism, the B-oligomer alone has also been shown to enhance glucose oxidation and glycerol release [16].

As such, lipolysis in isolated adipocytes and erythrocyte and platelet aggregation methods could provide ex vivo means to study specific properties of PTx, although these methods require isolation of primary cells from laboratory animals and humans. In addition, the low sensitivity of platelet aggregation needs to be considered.

## 3. Biochemical Assays

Biochemical assays for the detection of PTx determine either the enzymatic activity of the S1 subunit or the binding activity of the B oligomer. Although these two activities are considered as mutually exclusive within the toxin, PTx requires both functions, along with a translocation activity, in order to confer its toxicity inside cells. The loss of any of those three functions would render the holotoxin inactive. The binding and enzymatic functions do not require the dissociation of the toxin subunits in order to detect their individual activities in biochemical assays.

The binding and enzyme regions of PTx are impacted differently by the chemical inactivation processes commonly used in the manufacture of aP vaccines. Inactivation with glutaraldehyde causes crosslinking of lysine residues which are present in the B oligomer but not in the S1 subunit [114,115]. Therefore, glutaraldehyde reduces PTx binding capacity and prevents cell entry, but has little effect on its ADP-ribosylation activity [116]. In contrast, formaldehyde affects N-terminal amino groups of all PT subunits, which include arginine, cysteine, histidine, and lysine residues [117]. As these residues are present in both the S1 and B subunits, PTd made using formaldehyde-inactivation processes have reduced binding and enzyme activities [116].

Both of the biochemical assays described below are considered as potential alternatives to the *in vivo* models used for testing of aP vaccines and PTx products; however, a correlation between the enzyme or binding assays with the *in vivo* models has never been clearly demonstrated. The independence of these two biochemical functions, along with the differing susceptibilities of the subunits to the types of chemicals used in their inactivation further complicates establishing such a correlation. Moreover, the *in vivo* responses–either in the HIST or MWG test–are as much a reflection of complex biological effects that occur downstream from the initial Gα_i_ protein ribosylation or receptor activation caused by PTx and may be multifactorial. Therefore, the *in vivo* responses are much more variable in their outcome than are the biochemical assays which each assess an individual biochemical function of the PTx molecule. From a regulatory perspective, the attempts to establish direct correlations between the *in vitro* assays and *in vivo* models are not necessary and provide little assistance in the adoption of the animal-free methods as an alternative to the quality control of aP vaccines. Instead, the biochemical methods should be considered as an alternative approach for monitoring the production of inactivated PTd, which allow for assessment of the level of residual enzyme or binding activities that are unique to each manufacturing process.

### 3.1. Enzymatic-HPLC-Coupled Assay

In cells, the ADP-ribosylation by PTx normally occurs on the α_i/o/t_ subunit of the heterotrimeric G protein complex. ADP-ribosylation occurs uniquely at the cysteine residue of the α subunit protein located four amino acids from the carboxyl terminus [118]. Conformational changes conferred on the structure as a result of ADP ribosylation is likely important for the inactive state of the α subunit [119]. The S1 subunit of PTx was found to recognize a short synthetic peptide substrates that mimic the carboxyl terminus of Gα_i/o_ [120]. Based on these findings, Cyr et al. [121] developed an assay which uses a fluorescent-tagged substrate (HCAM-1) of 20 amino acids length which contains the requisite cysteine near the C-terminus. Ribosylation of this substrate in the presence of NAD^+^ and ATP can then be detected using standard HPLC techniques with a fluorescence detector. Under these incubation conditions [121], the PTx enzyme activity remains functional over at least a 24 h period with the longer incubation times improving the assay sensitivity to 20 ng/mL of PTx reference standard 90/518 (Table 4). This method is linear between 0.0625 and 4.0 µg/mL when duration of the reaction was 5 h and 15.6–500 ng/mL when the reaction was continued for 24 h [121]. The method can also be used directly to test for residual ADP-ribosylation activity in aP vaccines without the need for their pre-treatment or desorption [121,122]. As some inactivation procedures (partly) render the enzymatic activity of PTx unaffected, these vaccine preparations inherently possess a high background enzymatic activity, making small amounts of PTx difficult to detect in those samples [123]. A modification of the assay was later published to simplify the method by eliminating the need for heating the HPLC column and using an ammonium acetate running buffer to reduce precipitation issues [122]. The enzyme-HPLC method has been used in several international collaborative studies on the transferability of this assay and for the calibration of reference standards [123,124,125] and was shown to be reproducible across laboratories. Although the enzymatic-HPLC-coupled assay is promising, it can only monitor enzyme activity and the relatively high background enzymatic activity inherent to some PTd preparations might hamper the detection of low levels of PTx. 

### 3.2. Fetuin Binding Assay

PTx has two separate binding domains contained within the S2 and S3 subunits of the toxin, but a specific PTx receptor has not been identified [135]. Instead, PTx binds to the polysaccharides of various membrane-bound glycoproteins with affinities that vary depending on the glycan structures [136], with a preference for proteins with sialylated multiantennary N-glycans [126].

Gomez et al. (2006) first described a capture-ELISA method in which the sialylated protein fetuin, is used to coat microwell assay plates which are then incubated with purified PTx or desorbed aP vaccines. Any bound PTx or PTd is then detected by the use of polyclonal sheep IgG and HRP labelled secondary antibody. The assay is highly sensitive, being able to detect amounts as low as 2 ng/mL PTx (NIBSC reference preparation Code 90/518). For use in testing vaccines for potential residual PTx, the samples must be desorbed of any aluminium adjuvant in order to avoid interference with the assay. However, the high concentrations of PTd (5–40 µg/mL) which binds to fetuin with low affinity, makes it difficult to differentiate low levels of active PTx in the presence of such high amounts of PTd. Nevertheless, PTd affinity for fetuin was reported to be much more sensitive to changes in the binding buffer stringency (e.g., buffer ionic strength and pH) than was PTx, thereby allowing some increase in the specificity of the assay between bound PTx and PTd [127]. Specificity for PTx could be further enhanced with the use of a monoclonal antibody specific for an epitope that was modified by the chemical detoxication processes. Under these conditions it was possible to detect PTx at concentrations as low as 8 ng/mL when added into an aP vaccine made with formaldehyde-inactivated PTd [127].

## 4. Cellular Assays

**Figure 3:**
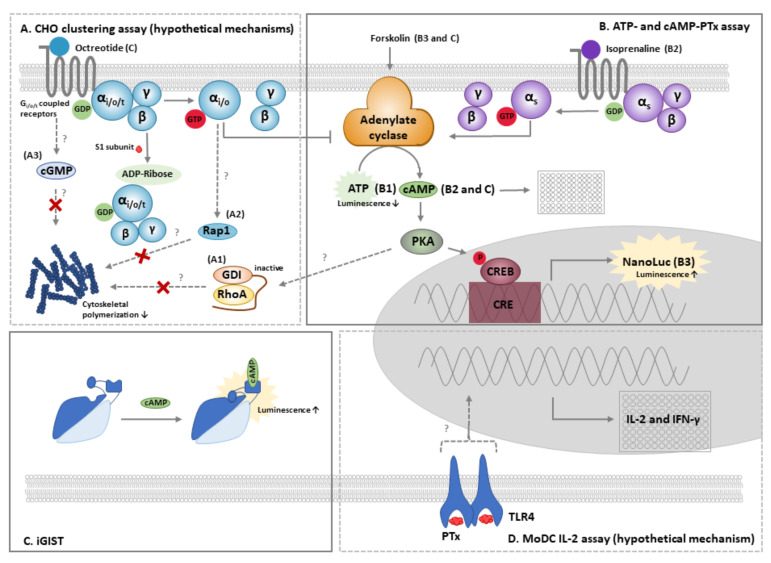
The cellular *in vitro* assays for PTx and their mechanisms. (**A**) The CHO cell clustering assay is based on the clustered growth pattern of CHO cells in response to PTx and requires an active S1 subunit. The morphological changes require rearrangement the cytoskeletal structures, but the underlying responsible mechanisms have not been fully elucidated. The first proposed mechanism (A1) involves the RhoA pathway, which normally results in polymerization of actin filaments. cAMP-dependent activation of PKA might result in an inactive state of RhoA, thereby diminishing or preventing actin polymerisation. Alternatively (A2), PTx-induced uncoupling of α_i_ proteins might directly affect the functioning of Rap1 and thereby reduce polymerization of cytoskeletal structures. In the third proposed mechanism (A3), uncoupling of Gα_i/o_ protein causes cGMP accumulation which also suppresses actin polymerization, affecting both cell shape and motility. Additional studies will be required to determine the involvement these three mechanisms in the CHO cell clustering assay. Clustering can be analysed manually and quantified by electrical impedance, continuous phase-contrast imaging or by measuring the distance between nearest neighbouring nuclei. (**B**) The ATP and cAMP PTx assays are based on the effects of PTx on the conversion of ATP to cAMP by the ACs. In the ATP-PTx assay (B1), the effect of PTx on ATP levels in peripheral blood mononuclear cells is measured using an ATP-luminescence assay. The cAMP-PTx assay is based on the rise in cAMP upon exposure of cells to PTx. Initially, A10 cells (B2) were used to monitor for PTx in combination with isoprenaline as an activator of AC using and a commercially available ELISA kits to measure cAMP levels. Subsequently, a CHO cell line stably expressing a CRE controlled NanoLuc construct was developed (B3). Upon stimulation with forskolin, PTx enhances the production of cAMP, leading to the activation of PKA, resulting in enhanced transcription of NanoLuc. The iGIST assay (**C**) is based upon HEK293 cells that co-express the Gα_i_-coupled SSTR2 receptor and a luminescent cAMP probe. In combination with octreotide and forskolin, these cells allow for real time assessment of cellular cAMP levels. PTx ADP ribosylates the α_i_ subunit of SSTR2, diminishing the effect of octreotide and results in enhanced levels of cAMP. cAMP binding to the probe causes a conformational change and increases the luminescence signal. In the MoDC assay (**D**), the effect of PTx on the production of the cytokines IL-2 and IFN-γ by MoDC is assessed. As PTx is described to stimulate the TLR4 receptor, this pathway may be involved in MoDC activation.

Various cellular assays for the detection of PTx have been developed, most of which rely on the PTx-dependent ADP ribosylation of the Gα_i/o_-coupled receptors and some on ADP ribosylation independent effects (see Figure 3 and Table 4). Although cellular assays are inherently more variable than biochemical assays, they harbour the potential to assess for whole PTx function, including translocation activity. As such, they are considered more informative of the physiological function of PTx, though standardization and validation is essential for successful use of the assays, especially when applied as a quality control measure in vaccine testing. 

### 4.1. The CHO Cell Clustering Assay

The CHO cell clustering assay is the most recognized and applied *in vitro* method for the study of PTx activity. The assay was first described by Hewlett et al. in 1983 [137] and is based on the clustered growth pattern of CHO cells when exposed to PTx. This morphological change requires rearrangement of cellular filament structures, but the underlying responsible mechanisms have not been fully elucidated. The involvement of the S1 subunit in clustering was demonstrated by two lines of evidence. Firstly, CHO cells expressing solely the S1 subunit exhibited a clustered growth pattern [138], while CHO cells exposed to the isolated B oligomer did not cluster at a concentration similar to the holotoxin [139,140]. Secondly, clustering did not occur upon exposure to a mutant form of PTx expressing an inactive S1 subunit [141,142]. Together, these findings suggest that clustering primarily relies on the A-subunit, although binding and internalisation is a prerequisite for the A subunit to reach the cytosol and execute its effect. However, exposure of CHO cells to PTx has particularly limited or no effect on cAMP levels, suggesting that clustering occurs as a result of minute changes in cAMP levels or as a result of a cAMP-independent mechanism. There are several potential signal transduction pathways which may lead to the clustering response of CHO cells by PTx (Figure 3A), of which one is the cAMP-induced PKA-RhoA pathway. This pathway was reported to be involved in cAMP-induced cell rounding of cardiac fibroblasts [134] and the shape of a variety of cell types including neuronal cells, macrophages, epithelial cells, endothelial cells, astrocytes, lymphocytes, mast cells, and platelets [143]. Therefore, it may also underly the PTx-induced change in CHO cell morphology. Alternatively, PTx-induced uncoupling of α_i_ proteins might directly affect the functioning of Rap1GAPII (a direct interactor protein of α_i/o_) and Rap1 and cause rearrangement of the cytoskeletal structure. PTx has been shown to interfere with this pathway and reduce neurite outgrowth [144]. Another mechanism has been postulated by Zamith et al. (2021), who suggested that CHO cell clustering may result from PTx-induced uncoupling of Gα_i/o_ protein causing inhibition of cGMP inactivation and suppression of actin polymerization, affecting both cell shape and motility [141]. Although all three hypotheses are feasible, further investigation will be required to determine their involvement in the CHO cell clustering assay.

Within the CHO cell clustering assay, CHO cells are exposed to a range of dilutions of the sample under study and the greatest dilution that results in clustering provides a semi-quantitative estimate of the PTx concentration. However, the assay has long suffered from inter-laboratory variability due to large differences between assay protocols [21,27,145,146]. Indeed, one study reported the inter-laboratory variability to be greater for the CHO cell clustering assay than the HIST [146]. This variability was substantially reduced when standardized procedures, cells and reagents were established for the assay [125]. As exposure of CHO cells to aluminium salts commonly found in aP vaccines disrupts their adherence and growth, the assay has mostly been used for calibrating purified PTx, monitoring the detoxification of PTd, and for testing other aP vaccine substances for trace PTx prior to the addition of the adjuvants [24]. Two strategies—direct and indirect—have been developed to overcome the problem of adjuvant interference in the assay. In the direct method, the aP vaccine samples are diluted sufficiently to negate the adverse impact of the vaccine adjuvant but the dilution should still be within the required detection limits of potential residual PTx. In the indirect method, the presence of any residual PTx content of a vaccine is monitored using semipermeable membranes to prevent direct contact between the cells and the aluminium salts [125]. The indirect method uses only the pelleted fraction of the aP vaccine so as not to dilute the culture media with the vaccine buffer, and assumes that the majority of residual PTx, if present, would be adsorbed to the aluminium. When both methods were evaluated in multi-laboratory collaborative studies, cytotoxicity from the adjuvant at the greater dilutions was still observed by most labs when using the direct method and when sufficiently diluted, the PTx concentration was too low to be reflected in a clustered growth pattern. However, the majority of the participants were able to detect a PTx spike added to the test aP vaccines when using the indirect method [125]. The level of sensitivity of the indirect method was considered similar to that of the HIST (Table 4). One criticism of the indirect method is that only the pellet fraction of the vaccine is tested and that any residual PTx in the aqueous fraction would be missed. However, it would be possible to measure the aqueous component using the fetuin-binding and enzymatic-coupled HPLC assays discussed above. In general, however, the majority of PTd and PTx quickly adsorbs to the aluminium salts in vaccines, although the extent can differ between adjuvant types and vaccines [130]. Therefore, the adjuvant fraction is considered the most relevant portion of the vaccine and allows for assessment of the maximum amount of PTx.

One of the major criticisms of using the CHO cell clustering assay is the manual interpretation of the morphological changes to the cultures, which is not only time consuming, but may cause some variability in assay outcomes. The identification of a clustered morphology and the final cut-off titre is a subjective decision made by the technician. In order to reduce this variability in assay outcomes [145], two technicians are recommended for scoring the clustering in the standardized method where at least one of those technicians is blinded to the sample identification [29,147]. Despite attempts by several research groups to quantify cell clustering induced by PTx for many years [148,149], only recently, three of such methods have been developed which allow for an objective measurement. In the first method [131], continuous phase-contrast imaging is used to monitor cellular confluency. In the study, minor effects of PTx on cellular confluency were already noticeable at 1 ng/mL PTx, while effects were pronounced from 10–1000 ng/mL PTx [131]. A second method uses the xCELLigence™ Real-Time Cell Analysis system, which measures the electrical impedance as a result of cell attachment and proliferation. As incubation of CHO cells with PTx results in reduced growth and increased rounding, the impedance is reduced upon exposure to PTx between 23–5803 mIU/mL PTx (BRP1, CHO units) and in PTx-spiked genetically detoxified PTx (dPTx) [132]. However, its application for aP vaccine testing is limited by the method’s inability to differentiate between PTx-induced clustering and cytotoxicity which might occur from other vaccine components. The third method measures the distance between nearest neighbouring nuclei of CHO cells, a parameter that reflects the growth pattern of the cells. The distance between each nucleus and its most adjacent nucleus was decreased by purified PTx at concentrations of 3–725 mIU/mL (BRP1, CHO units) (BRP1) and 0.005–4 ng/mL (LIST Biological), as well as in the context of two aP containing vaccines (45–181 mIU/mL BRP1, CHO units) (Hoonakker et al. submitted). Although the last method seems to be more sensitive than the other two, the application of each of these methods significantly improves the assessment of CHO cell clustering and might consequently foster application and implementation of CHO cell clustering assay for both research-oriented questions, as well as vaccine QC and lot release purposes. 

### 4.2. The ATP and cAMP-PTx Assay

Since the majority of the effects of PTx on cellular functions are associated with the cAMP-PKA pathway, assays have been developed to measure these effects directly with the intent to provide a more defined and quantitative assessment of PTx activity. The effects of PTx on the conversion of ATP to cAMP by the ACs have been studied by measuring both the decline and the increase in cellular cAMP and ATP, respectively (Figure 3). Dr. Bache developed a method based on the effect of PTx on ATP levels in peripheral blood mononuclear cells [123]. The assay is easy to perform, fast, and inexpensive and was demonstrated to detect 0.36–15 IU/mL PTx (BRP1, CHO units), but this sensitivity was considered insufficient for requirements in the testing of aP vaccines for lot release purposes [123]. In contrast, we developed an assay based on the rise in cAMP upon exposure of cells to PTx [130,150]. Initially, A10 cells were used to monitor for PTx in combination with isoprenaline as an activator of ACs [150]. Isoprenaline is a β-adrenergic receptor agonist and activates Gα_s_ and thereby stimulating ACs to produce cAMP. Using this method, the effects of 25–200 ng/mL purified PTx (Sanofi Pasteur, Toronto, ON, Canada) on intracellular cAMP levels were detected by assessing cAMP in cell lysates using commercially available ELISA kits. The assay was successfully transferred between two laboratories; however, it suffered from reproducibility and sensitivity problems. In response to these issues, cAMP reporter cell lines were developed using CHO and A10 cells. The cells were stimulated to produce cAMP using isoprenaline, norepinephrine (α/β-adrenergic receptors agonist) or forskolin. Since the response of A10 cells to forskolin were most pronounced and CHO cells only responded to forskolin, this stimulator was selected for both cell lines. The CHO and A10 reporter cell lines detected PTx in a concentration-dependent manner with a limit of detection of 36 mIU/mL PTx (BRP1, CHO units) [130]. By exposure to the pellet fraction of an aP vaccine spiked with PTx and using semi-permeable inserts, CHO and A10 cells detected as low as 181 and 363 mIU PTx/mL (BRP1, CHO units), respectively, although the responses by the CHO reporter cells were more pronounced. Additional experiments were performed to optimize conditions for direct exposure of the CHO reporter cells to the pellet fraction of aP vaccines. By applying these conditions, the performance of the CHO reporter cell lines was analysed in two pre-validation studies using purified PTx and PTx spiked into two commercially available aP vaccines (Hoonakker et al. in progress). These studies showed appropriate linearity, accuracy, and robustness for purified PTx and PTx spiked aP vaccines. The limit of detection was 23 and 68 mIU/mL and the linear range was 23–136 mIU/mL and 68–363 mIU/mL (all BRP1, CHO units) for purified PTx and PTx spiked into two aP vaccines, respectively. The CHO reporter cell lines were also used in a collaborative study for the establishment of the WHO 2nd IS for PTx [29]. The activity of the new standard relative to JNIH-5 for PTx measured with the CHO reporter cells was comparable to the activity assess by the CHO cell clustering assay [29]. This underlines the potential of the cAMP-PTx assay as an very promising, qualitative, and sensitive assay for detection of PTx activity.

### 4.3. Other Cellular Methods

ADP ribosylation assays that assess the addition of an ADP ribose to α_i/t/o_ in cell membranes have been widely used in studies into the cellular effects of PTx [6,7,151]. Early assays monitored ADP ribosylation by PTx using radio-labelled ^32^P-NAD^+^. However, in the interest of occupational health and safety concerns, alternative methods with non-radioactive substrates should be considered. Existing and novel radioactive-free methods for ADP-ribosylation have been reviewed by van der Heden van Noort [152]. In the classical signal transduction model, ADP ribose of α_i/o/t_ proteins affects AC functions, thereby resulting in the accumulation of cAMP. Like ADP ribosylation, changes in cAMP levels were historically determined by radioimmunoassays, while nowadays many radioactive-free methods are available [153], some of which allow assessment of cAMP levels in living cells [154]. One is the 22F cAMP probe developed by Promega. This probe has been utilized by Paramanov et al. [131], who developed a method based on HEK293 cells that co-express somatostatin receptor 2 (SSTR2), and the luminescent cAMP probe. In combination with forskolin, these cells allow for real time assessment of cellular cAMP levels. This cell system was developed since SSTR2 belong to the α_i_ GPCR family, which activity can be well controlled by the specific ligand octreotide. The sensor cells have been successfully applied for screening of nanoparticle-ligand complex binding to SSTR2 [155] and was able to detect 1–1000 ng/mL of PTx (LIST Biological and Invitrogen) and 100 ng/mL of PTx in the context of a commercial aP vaccine. However, as the sensor cells were highly sensitivity to certain compounds, such as organic solvents and alcohols, a strict parallel design method—including all solvent dilutions—is required. This potentially makes data processing highly complex. In that context, the effect of the unspiked vaccine on cAMP levels were predictable; however, the absence of matched controls hampered the evaluation of the responses. Taken together, the sensor cell method is an extremely precise tool for PTx research, but data processing requires automation and the potential of the method for vaccine batch release testing would require further studies and evaluation.

The cellular effects of PTx described above are primarily the result of the intracellular ADP-ribosylation activity of the S1 subunit. Although ADP-ribosylation occurs rapidly and are apparent even at particularly low concentrations of PTx [39,156], various B-subunit ADP-ribosylation-independent effects have also been described [156]. A broad analysis of the effect of PTx have been performed using genetic assays. DNA array analysis of the effects of PTx in lung tissues of rats identified various potential biomarkers [157,158]; however, additional studies into these biomarkers were not performed. PTx effects were also detected in *in vitro* cultured human monocyte derived dendritic cells (MoDC) [133] and resulted in the identification of IL-2 and IFN-γ as the most promising parameters. Although IL-2 and IFN-γ are cytokines predominantly produced by T cells and natural killer cells, recent studies show that MoDC modulate the immune response by producing IL-2 [159,160] and IFN-γ [161]. The biomarkers IL-2 and IFN-γ were affected by PTx concentrations as low as 12.5 IU/mL (JNIH-5) and 100 ng/mL (GSK). The suitability of this method for vaccine purposes however awaits further studies.

In addition to these *in vitro* assays, many other animal-free methods have been utilized to study PTx. The properties studied often reflect *in vivo* mechanisms or details of these mechanisms. As such, immune modulation has been investigated with human MoDCs, showing that PTx, its B oligomer [19,20] and dPTx [20,162,163] induce maturation and the release of pro-inflammatory cytokines, such as IL-6 and IL-12p40 [19,164]. Details of the signalling pathway have been investigated with the human cell line HEK293, that stably expresses TLR4 or TLR2. These cell lines demonstrated that PTx and dPTx can bind to TLR4 and induce cell signalling through NF-κB [19,162], while TLR2 was only activated by dPTx [162]. PTx-induced signalling through NF-κB was confirmed in cardiac fibroblasts, cells that increase the expression of IL-1β in a NADPH oxidase-dependent manner [20]. However, the role of the enzymatic activity in maturation of DCs is uncertain. While one study showed that maturation of dendritic cells and MoDC is induced by dPTx but not by formaldehyde-inactivated toxin [164], another study reported that neither the B oligomer nor dPTx was capable of exerting such an effect [165]. Although these studies provide proof for the effect of PTx on TLR signalling and antigen presenting cell activation, one study showed that LPS contamination of some PTx preparations can be responsible for the observed effect [166]. Especially when high concentrations of PTx are used, the LPS contamination should be controlled for, which has been done in the studies referred to above.

The capacity of the PTx and dPTx on T cell responses have been studied by co-culturing purified allogeneic T lymphocytes with MoDCs [19,162,163]. These studies demonstrate that PTx induces T cell proliferation [19] and that both PTx and dPTx increase the levels of the Th1 cytokine IFN-γ [162,163], while dPTx results in enhanced levels of the Th17 cytokine IL-17 and inhibits the release of the Th2 cytokine IL-5 [162]. Finally, there is an *in vitro* assay based on the effect of PTx on T cell migration, assessing the penetration of T lymphoma cells through a monolayer of fibroblast-like cells. In this model, PTx inhibits the migration of T cells in a concentration-dependent manner between 10^−14^ –10^−12^ M PTx [167]. The data indicate that the migration of T cells responded only to the holotoxin, while the isolated B oligomer and the A protomer were both ineffective. Similarly, the effect of PTx on neutrophil recruitment has also been demonstrated *in vitro* using a human neutrophil chemotaxis assay with 48-well plates fitted with microchemotaxis chambers and transwell membranes. In this system, chemotaxis of the cells towards formylmethionyl-leucyl-phenylalanin and zymosan-activated human serum was inhibited in concentration-dependent manner (10–250 ng/mL PTx) [168].

Based on the effects of PTx on the infiltration of immune cells in the CNS, the *in vitro* ‘human brain microvascular endothelial cell barrier model’ was developed. The model employs the human brain-derived microvascular endothelial cell line HBMEC in a two-compartment system and measures the transendothelial resistance and permeability. PTx (200 ng/mL) but not its B oligomer was shown to enhance the “vascular” permeability and to increase the migration of monocytes [169]. Not surprisingly, the endothelial cells showed extensive ADP-ribosylation upon exposure to PTx. Effect of PTx on tumor cells have also been studied in cell migration assays. Using the bladder cancer cell line J82, PTx (0.01–100 ng/mL) was shown inhibit the migration of the cells towards lysophosphatidic acid [91]. Two other assays were developed by Gilder et al. that use high grade glioma-like cell lines and assess their migration through a transwell membrane and into a matrigel. PTx inhibited both cell migration (10–1000 ng/m) and invasion (1 µg/mL) [170].

## 5. Regulatory Considerations of PTx Testing

Monitoring for potential trace levels of residual PTx activity in aP vaccines is a standard control test for ensuring the consistent production of safe and effective vaccines which include chemically inactivated PTd. Active PTx levels can be monitored during and/or following the chemical inactivation step to ensure its completeness. This is usually done prior to the addition of any adjuvant so as to minimize its interfering with test methods. Two advantages to monitoring at this stage are that the PTd is at a greater concentration than is present in a formulated vaccine product, and that the highly sensitive *in vitro* methods can be used to test the bulk substance due to the absence of an aluminium adjuvant(s).

Although testing of PTd samples prior to the adsorption step has its advantages, most national regulatory authorities require assurance of safety following the formulation and filling of the vaccine vials in addition to all other tests for quality and potency. Stability testing throughout the shelf-life of the product is also expected. As the presence of aluminium adjuvants may cause interference with some of the *in vitro* assays, the test for residual PTx in the final product is traditionally performed using one of the HIST methods. Although differences between regulatory requirements on how the test is performed can influence the assay sensitivity, the estimated IC_50_ and IC_5_ have been calculated at approximately 20 and 2 IU PTx (HIST units) of the BRP1 preparation, respectively, for the method commonly used in Europe [28]. As the standardized CHO cell clustering assay described above was found to have a limit of detection of approximately 5 mIU of CHO cell units/mL (BRP1) when using the same PTx preparation, its sensitivity is well within consideration if it is used as an alternative to the murine HIST method.

## 6. Concluding Remarks

Although the most important area PTx use is testing of aP vaccines, this toxin has been valuable in many other research areas because of its highly potent inhibitory effect on Gα_i/o/t_ proteins. All the *in vivo* models and *in vitro* assays addressed in this review have the potential to identify PTx, but differ with respect to their nature, the measured properties, sensitivity, cost and lead time. For the *in vivo* models, animal welfare concerns also play an important role as especially the HIST is associated with a high level of pain and distress. Furthermore, the presence of a matrix is incompatible with some of the methods, affects the signal, or requires the inclusion of adequate controls. The relationship between PTx and outcome of the method might be nonlinear, and some of the methods suffer from subjectivity. Consideration for the choice of the method should include these factors, in conjunction with the specific requirements for the purpose of the study. As this review provides an overview of the existing *in vivo* and *in vitro* methods, it might aid to compare the methods and choose the most appropriate. Many of the reviewed *in vitro* methods are mechanistically well founded as opposed to most of the *in vivo* methods, and allow for the detecting of lower levels of PTx. This minimizes the risks and should encourage the adoption of *in vitro* methods for regulatory and non-regulatory purposes. 

## Figures and Tables

**Table 1:** Dose-toxicity response for PTx administered to humans and mice.

	PTx Dose	PTx Source	Response	Reference
In adults	1.0 μg/kg	*B. pertussis* Tohama	no adverse effects	[17]
In children	260 to 300 ng	PTx in two wP vaccines	considered safe for vaccination	[24,25]
In mice	200 ng	purified from *B. pertussis* 3779	no deaths	[26]
In mice (HIST) *	12 IU **	BRP1 (HIST)	ED_50_	[27]
In mice (HIST) *	1–2 IU ***	BRP1 (HIST)	ED_5_	[28]

* Dose that sensitizes mice to histamine. ** Corresponding to approximately 78 ng. *** Corresponding to approximately 6.7–13 ng.

**Table 2:** Commonly used PTx preparations and their potencies.

Name	Provider	µg/Vial	IU/Vial HIST	IU/Vial CHO
JNIH-5	WHO	62.5 *	10,000	10,000
2nd IS	WHO	20	1881	680
BRP1	EDQM	50	7500	1360
BRP2	EDQM	n.d.	n.d.	130
LIST Biological	LIST Biological	50	n.d.	n.d.

* protein nitrogen content of 10 µg. n.d. not determined.

**Table 3:** Common *in vivo* models and their characteristics.

Model	PTx Source and Detected Range			PTx Range Detected in Vaccines	Coverage of PTx Properties and/or Mechanism	Main Area of Application *	Ref.
	1	2	3				
HIST lethal pass/fail	2–12 IU (HIST) BRP1 **	2–15 ng LIST Biological	5–125 ng U.S. PT control preparation	5–74 ng/mL *** (wP vaccine), 2–15 ng LIST Biological	Binding, internalisation, and ADP ribosylation	Release testing and research	[24,27,33,43,47]
HIST temperature pass/fail	1.5–7.5 IU (HIST) BRP1				Binding, internalisation, and ADP ribosylation	Release testing and research	[44]
HIST temperature quantitative	1–4 HSU/mL of Japanese ref. aP preparation	0.58–5.25 IU (NIBSC 90/518)		0.01–1 IU ^§^ (aP vaccine)	Binding, internalisation, and ADP ribosylation	Release testing and research	[45,46]
LP	20–4000 ng (NIH 114 (3779B)) ^§§^	188–1500 ng (JNIH-5)			Binding, internalisation, and ADP ribosylation	Release testing and research	[48,49,50]
MWG	113–450 ng (W28)	4000 ng ^§§^ (strain n.s.)	375–1500 ng (JNIH-5)	65–370 ng *** (wP vaccine)	Unknown	Release testing of wP vaccines and research	[24,40,41,50]
IAP	8–2000 ng (Tohama) ^§§^				Binding, internalisation, and ADP ribosylation	Research	[42]
Vascular permeability	1–100,000 ng (Tohama) ^§§^				B oligomer-dependent	Research	[51,52]

* Test methods used for lot release are not necessarily legislated by regulators but need to be validated by each vaccine manufacturer, and are accepted/rejected based on the scientific evidence presented within a vaccine licensure application or a change of this application. ** LD_5_–LD_50_. *** PTx content of wP vaccines determined with ELISA. ^§^ Estimates of PTx activity in various vaccine formulation expressed as IU of NIBSC (90/518). ^§§^ PTx was harvest and purified in house. n.s. not specified.

**Table 4:** Common *in vitro* assays and their characteristics.

Assay	Source of PTx and Detected Range			Detected Range in aP Vaccines	Coverage of PTx Properties	Compatible with	Main Area of Application *	Ref.
	1	2	3					
Fetuin ELISA	4–250 ng/well (NIBSC 90/518)	1.5–196 ng/mL (Sanofi Pasteur)		0.36–3.63 IU (CHO)/mL BRP1	Binding	Purified PTx and desorbed aP vaccine preparations	Research	[123,126,127]
HPLC	62.5–4000 ng/mL (NIBSC 90/518) ^a^	15.6–500 ng/mL (NIBSC 90/518) ^b^	10–100 ng (NIBSC 90/518) ^c^	0.5–2.25 µg/mL (NIBSC 90/518)	Enzymatic activity	Purified PTx and complete aP and wP vaccine preparations **	Research	[121,122,123,128]
CHO cell clustering (visual reading)	1.27–1813 mIU (CHO)/mL BRP1	1.14–8 mIU/mL JNIH-5	0.1–30 ng/mL LIST Biological	181–725 mIU (CHO)/mL BRP1 ***	Binding, internalisation, enzymatic activity	Purified PTx and pellet fraction aP vaccines	Bulk testing of aP vaccines and research	[47,125,129,130]
CHO cell clustering (confluence analysis)	1–1000 ng/mL LIST Biological				Binding, internalisation, enzymatic activity	Purified PTx	Research	[131]
CHO cell clustering (impedance)	23–5803 mIU (CHO)/mL BRP1	0.4–49 ng/mL (PTx Sanofi Pasteur)		453 and 1813 mIU (CHO)/mL BRP1 ^§^	Binding, internalisation, enzymatic activity	Purified PTx, PTd	Research	[132]
CHO cell clustering (3N method)	3–725 mIU (CHO)/mL BRP1	0.005–4 ng/mL LIST Biological		45–181 mIU (CHO)/mL BRP1	Binding, internalisation, enzymatic activity	Purified PTx and pellet fraction aP vaccines	Research	Hoonakker et al. submitted
cAMP-PTx reporter	23–136 mIU (CHO)/mL BRP1 (*linear range*)	25–1600 mIU/mL JNIH-5	5-160 ng/mL WHO 2nd IS 15/126	68–363 mIU (CHO)/mL BRP1 (*linear range*)	Binding, internalisation, enzymatic activity	Purified PTx and pellet fraction aP vaccines	Research	[130] Hoonakker et al. in preparation
iGIST	1–1000 ng/mL LIST Biological	1–1000 ng/mL Invitrogen		100 ng/mL LIST Biological	Binding, internalisation, enzymatic activity	Purified PTx and complete aP vaccines	Research	[131]
MoDC IL-2	12.5–50 IU/mL JNIH-5	100 and 250 ng/mL GSK			Unknown	Purified PTx	Research	[133]

* Test methods used for lot release are not necessarily legislated by regulators but need to be validated by each vaccine manufacturer and are accepted/rejected based on the scientific evidence presented within a vaccine licensure application or a change of this application. ** The background enzymatic activity is highly variable between vaccine preparations and small amounts of PTx might not be detected if the background enzyme level is already high. Therefore, the HPLC can be applied for monitoring of the consistent levels of the background enzyme activity. *** Only the indirect method is compatible with the pellet fraction of vaccines (Isbrucker et al. 2016 [134]). ^§^ Instead of vaccine, genetically detoxified PTx (Sanofi Pasteur Canada) was used. ^a^ 5 h incubation. ^b^ 24 h incubation. ^c^ 6 h incubation.

## Data Availability

Not applicable.

## Abbreviations

AC	adenylate cyclases
aP	acellular pertussis
BRP1	Biological Reference Preparation 1
CHO	Chinese hamster ovary
CRE	cAMP response elements
CREB	cAMP response element-binding protein
dPTx	genetically detoxified PTx
EAE	experimental autoimmune encephalomyelitis
ED	Effective Dose
EDQM	European Directorate for the Quality of Medicines & Healthcare
EPAC	ex-change protein directly activated by cAMP
FHA	filamentous hemagglutinin
HIST	histamine sensitization test
IAP	Islet-activating protein
IS	International Standard
IU	International Units
LP	Leukocytosis promotion
LPS	lipopolysaccharide
MoDC	monocyte derived dendritic cells
MS	multiple sclerosis
MWG	Mouse weight gain
NIBSC	National Institute for Biological Standards and Control
Ph. Eur.	European Pharmacopeia
PKA	protein kinase A
PTd	pertussis toxoid
PTx	Pertussis toxin
SSTR2	somatostatin receptor 2
WHO	World Health Organization
wP	whole-cell pertussis
